# A simplified point-of-service model for hepatitis C in people who inject drugs in South Africa

**DOI:** 10.1186/s12954-023-00759-0

**Published:** 2023-03-04

**Authors:** Elaine Saayman, Vanessa Hechter, Ndoliwe Kayuni, Mark W. Sonderup

**Affiliations:** 1 Sediba Hope Medical Centre NPC, Tshwane, South Africa; 2 Tshwane Project, Medecins Sans Frontieres, Tshwane, South Africa; 3 Division of Hepatology, Department of Medicine, Faculty of Health Sciences and Groote Schuur Hospital, University of Cape Town, Cape Town, South Africa

**Keywords:** Hepatitis C, People who inject drugs, Point-of-care, Harm reduction

## Abstract

**Background:**

Globally, 9% of people who inject drugs (PWID), a key hepatitis C-infected population, reside in sub-Saharan Africa. In South Africa, hepatitis C seroprevalence in PWID is high. It is almost 84% in Pretoria and hepatitis C genotypes 1 and 3 predominate. Access to hepatitis C care for PWID is inadequate given low referral rates, socio-structural barriers, homelessness and limited access to harm reduction. Traditional care models do not address the needs of this population. We piloted a simplified complete point-of-service care model, a first of its kind in the country and sub-continental region*.*

**Methods:**

Community-based recruitment from Pretoria’s PWID population occurred over 11 months. Participants were screened with point-of-care rapid diagnostic tests for HBsAg (Alere Determine™), hepatitis C and HIV antibodies (OraQuick®). Qualitative HCV viremia was confirmed on site with Genedrive® (Sysmex), similarly at week 4, end of treatment and to confirm sustained virological response. Viremic hepatitis C participants were initiated on 12 weeks of daily sofosbuvir and daclatasvir. Harm reduction and adherence support, through directly observed therapy, peer support, a stipend and transport, was provided.

**Results:**

A total of 163 participants were screened for hepatitis C antibody, and 66% were positive with 80 (87%) viremic. An additional 36 confirmed hepatitis C viremic participants were referred. Of those eligible to initiate treatment, 87 (93%) were commenced on sofosbuvir and daclatasvir, with 98% (*n* = 85) male, 35% (*n* = 30) HIV co-infected, 1% (*n* = 1) HBV co-infected and 5% (*n* = 4) HIV/HBV/HCV triple infected. Some 67% (*n* = 58) accessed harm reduction packs, 57% (*n* = 50) opioid substitution therapy and 18% (*n* = 16) stopped injecting. A per protocol sustained virological response of 90% (*n* = 51) was achieved with 14% (*n* = 7) confirmed reinfections following a sustained virological response. HCV RNA qualitative testing performance was acceptable with all sustained virological responses validated against a laboratory assay. Mild adverse effects were reported in 6% (*n* = 5). Thirty-eight percent (*n* = 33) of participants were lost to follow-up.

**Conclusion:**

In our setting, a simplified point-of-service hepatitis C care model for PWID yielded an acceptable sustained virological response rate. Retention in care and follow-up remains both challenging and central to success. We have demonstrated the utility of a model of care for our country and region to utilize this more community acceptable and simplified practice.

## Background

Globally, an estimated 58 million people are chronically viremic for hepatitis C virus (HCV) infection [1]. It is a major global public health concern. Key populations such as people who inject (PWID) and men who have sex with men are the major drivers of new hepatitis C virus infections [2]. To achieve hepatitis C elimination, key populations need treatment access, yet experience the greatest barriers to care [3]. PWID are also at risk of other blood-borne infections including hepatitis B and HIV but have, in general, less access to harm reduction programs. Globally, 9% of PWID reside in sub-Saharan Africa [4]. Other hepatitis C transmission risk in sub-Saharan Africa occurs in healthcare settings due to unsafe medical practices [5, 6].

Hepatitis C seroprevalence in the general South African population is low and estimated at 0.5% [UI 0.4–1.0] equating to between 226 000 and 572 000 chronically infected people [7]. There are sub-population pockets of significant infection in South Africa, expectedly in at-risk populations such as PWID and men who have sex with men. In Pretoria, ultra-high seroprevalence and viremic rates for hepatitis C have been demonstrated. Hepatitis C viral genotypes circulating in PWID in Pretoria, include genotypes 1 and 3 [8], although South Africa is considered a pan-genotypic country, with genotypes 1 through 5 demonstrated [9]. Sediba Hope Medical Centre, a non-profit primary healthcare facility, providing care to vulnerable populations in Pretoria’s central business district and surrounds, has observed an increasing trend of both HCV mono- and HIV–HCV co-infection [10]. The ability for these patients to access care is limited. This is due to both the relative unavailability of hepatitis C care and traditional care models, invariably facility-based specialist services at tertiary healthcare facilities. These specialist clinics do not specifically cater for the needs of PWID as a vulnerable and challenging population. PWID care is inadequate because of low referral rates [11], socio-structural barriers, homelessness [12] and limited access to harm reduction interventions [13]. Previous local PWID population screening serosurveys did not include significant successful linkage to care [10, 14].

The South African National Hepatitis Guidelines and costed Action Plan [15], as well as local Harm Reduction Guidelines [16], outline processes for eliminating viral hepatitis as a public health threat by 2030 [14]. The implementation and scale-up of these strategies has been delayed due to inadequate funding and the COVID-19 pandemic. Thus, harm reduction services have been provided predominantly through civil society [17], including Sediba Hope Medical Centre [18].

Simplified service approaches minimizing the time between screening and linkage to care have been modeled for viral hepatitis in PWID in Tanzania demonstrating cost-effectiveness and improved outcomes [19]. These approaches emphasize the use of rapid diagnostics, integration in harm reduction programs and population-sensitive monitoring modalities. To address our challenges, we designed and evaluated a simplified complete point-of-service care model for PWID to provide access and simplification of care. Our approach, a first of its kind in the sub-region, included a full point of access service utilizing Genedrive®, a point-of-care molecular hepatitis C RNA technology [20].

## Methods

**Fig. 1:**
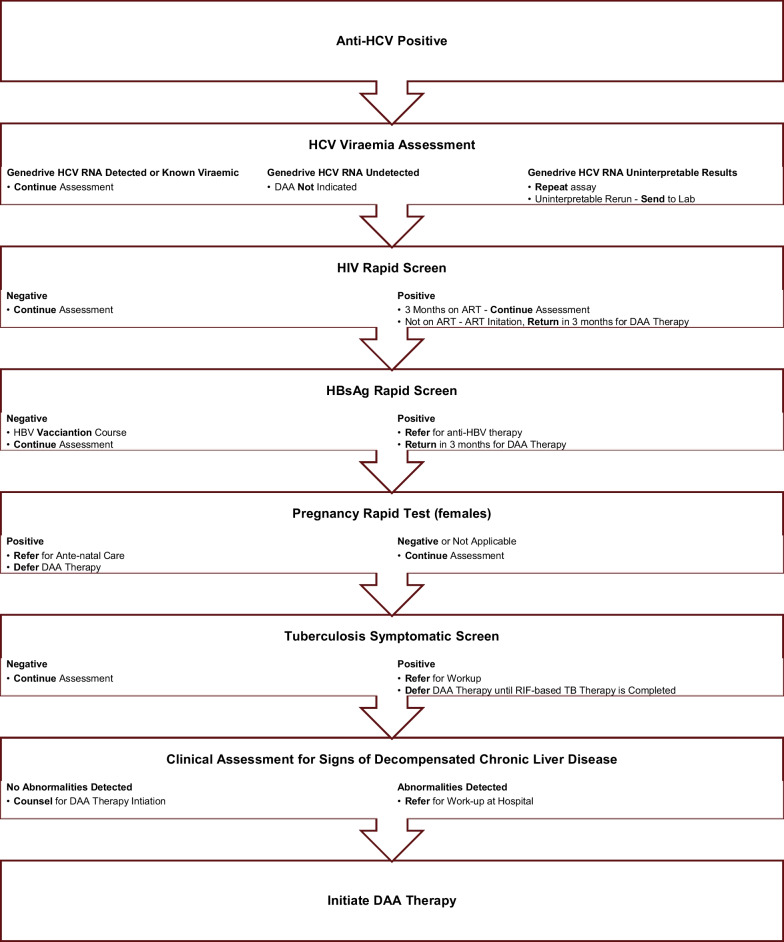
Enrollment procedure for simplified hepatitis C therapy. Anti-HCV = hepatitis C virus antibody; DAA = direct-acting antivirals; HBsAg = hepatitis B virus surface antigen; HBV = hepatitis B virus; HCV = hepatitis C virus, HIV = human immunodeficiency virus; Lab = laboratory; RIF = rifampicin; RNA = ribonucleic acid; TB = tuberculosis

A simplified community-based recruitment model linking Pretoria’s PWID population to hepatitis C treatment was initiated. Over the course of a year, PWID (includes those who inject and smoke heroin) were recruited. Recruitment strategies included sampling from existing primary healthcare programs, using available tracing data, and later applying snowball recruitment methods; “hot spot” outreaches; and direct communication with community program implementation partners. Recruited participants were screened for hepatitis B, C, HIV and tuberculosis. Point-of-care rapid diagnostics tests were used: hepatitis B surface antigen (HBsAg, Alere Determine™, USA), anti-HCV (hepatitis C antibody) and HIV antibody tests (OraQuick®, USA). These point-of-care tests were previously validated in a South African setting [21–23]. Participants were recruited if they met inclusion criteria: ≥ 18 years of age, were hepatitis C direct -acting antiviral therapy naïve (treatment experienced with PEG-interferon/ribavirin) and were able to complete, sign and date the approved informed consent. Pregnancy was an exclusion criterion. HIV co-infected participants stable on antiretroviral therapy for ≥ 3 months were included. Participants, with confirmed hepatitis C viremia and a new diagnosis of HIV (with or without hepatitis B), were initiated and stable on antiretroviral therapy for 3 months prior to hepatitis C direct-acting antiviral therapy. Those with hepatitis B (HBsAg positive) and HIV negative were excluded, unless stable on anti-HBV therapy. All HBsAg-negative participants were offered hepatitis B vaccination. Participants who screened sputum positive for tuberculosis were excluded and referred for further workup (Fig. 1).

Participants screening anti-HCV positive had qualitative HCV viremia confirmed using Genedrive® (Sysmex, South Africa). This was performed on-site at the point of service. The assay is a two-phase procedure requiring 30 µL of plasma or serum preparation (30-min centrifuge), mixed with 60 µL nuclease-free water, which is added to a three-channel cartridge per manufacturers’ instructions. This is followed by a reverse transcription reaction to generate complementary DNA, which runs for 88 min on the portable desktop Genedrive® device. Of four possible outcomes, hepatitis C RNA ‘detected’ (lower limit of detection > 2362 IU/ml) confirms viremia [20].

A clinical evaluation was performed to identify features of chronic liver disease. No formal evaluation of liver fibrosis or hepatitis C genotyping was performed at baseline. Hepatitis C viremic participants who met eligibility criteria were initiated on 12 weeks of daily sofosbuvir (400 mg) and daclatasvir (60 mg) as per South African National Viral Hepatitis Guidelines [15]. Where required, HIV co-infected participants had the daclatasvir dose adjusted to account for drug–drug interactions.

Harm reduction and adherence support, through directly observed therapy and peer support, was provided. Support allowances were offered to participants on a weekly scale to accommodate for transport and time, aligned to the South African Health Products Regulatory Authority (SAHPRA) guidelines for research participants [24].

Treatment monitoring at week 4, end of treatment and to confirm a sustained virological response, at 12 weeks post-end of treatment, was performed on-site with Genedrive®. A hepatitis C RNA reading as ‘undetected’ with Genedrive® was accepted as proof of sustained virological response. Samples were validated with a quantitative hepatitis C RNA at a reference laboratory [25]. Suspected reinfections at 12 weeks post- end of treatment were confirmed with hepatitis C genotype and molecular testing in a post hoc analysis.

Data from testing logs, participant files and laboratory results were captured using Microsoft Excel (version 10) and stored on a password-protected database, accessible only to the authorized study team, in compliance with local regulations and legislative requirements. Standard per protocol descriptive analyses and inferential statistics (Pearson’s Chi-squared test) were generated in Stata17 (Stata Corp., USA) from demographic data, participant case tracking, treatment outcomes and support services, to assess the program’s efficacy.

The study was ethically approved (UCT Human Research Ethics Committee R045/2014; 793/2022). Prior to enrollment or screening, all participants received an informed consent outlining the research aims, target population, eligibility criteria, participant rights and confidentiality, all explained in a language of their choice.

## Results

**Fig. 2:**
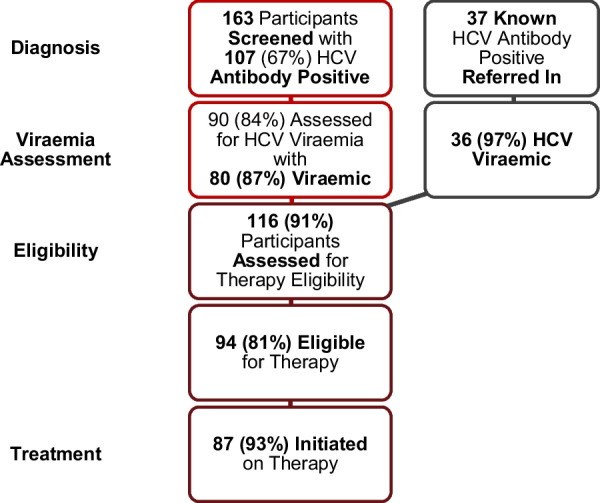
Enrollment assessment for hepatitis C therapy. HCV =  hepatitis C virus

From mid-2020 to the first quarter of 2021, 163 participants were screened. Two-thirds, 66% (*n* = 107), screened anti-HCV positive. Of them, 42% (*n* = 59) were HIV co-infected. HBsAg was positive in 2% (*n* = 6). Of the 107 participants who screened anti-HCV positive, 84% (*n* = 90) were confirmed active hepatitis C viremia. The remainder, *n* = 17, declined or defaulted before a blood draw for confirmation could occur. Of the 90 participants who underwent confirmation for the presence of hepatitis C RNA viremia, 87% (*n* = 80) were positive and linked to care (Table 1). During the screening period, an additional 37 participants presented for care when hearing about the treatment project underway. Most of these participants, *n* = 29, had previously screened positive and were actively viremic, but untreated. Among the remaining untested 8, *n* = 7 (88%) were viremic. Accordingly, in total, 116 participants were confirmed HCV viremic. In terms of treatment eligibility, 81% (*n* = 94) met criteria. The remaining 22 participants were ineligible for the following reasons: not stable on antiretroviral therapy (*n* = 18, 82%) or anti-HBV therapy (*n* = 1, 5%), declined treatment (*n* = 1, 5%) and loss to follow-up (2, 9%). Among the 94 treatment eligible participants, 87 (93%) initiated direct-acting antiviral therapy (Fig. 2), *n* = 6 defaulted before starting treatment and one other withdrew consent. The majority (*n* = 52, 60%) of the treatment-initiated cohort (started on direct-acting antiviral therapy) had hepatitis C mono-infection, 35% (*n* = 30) were HIV co-infected, 1% (*n* = 1) HBV co-infected and 5% (n = 4) triple infected. Demographic parameters of participants are listed in Table 2, with most participants male (*n* = 85, 98%) and of black African ethnicity (*n* = 63, 72%). Median age was 34 years [IQR 31–41]. All participants were current or previous heroin users, with most, *n* = 80 (92%), unaware of their injecting partners’ hepatitis C status.

**Table 1:** Recruitment and screening

Recruitment and screening	*n*	%	*n*	%	*n*	%	*n*	%
Recruitment method	Point-of-care screening		Traced		Referred In		Total	
HCV								
Anti-HCV assessment	163						**163**	
Anti-HCV positive	107	65.6%	29		8		**144**	
HCV viremia assessment	90	84.1%	29	100.0%	8	100.0%	**127**	**88.2%**
HCV detected, i.e., viremic	80	87.0%	29	100.0%	7	87.5%	**116**	**91.3%**
HCV RNA undetected	8	8.7%			1	12.5%	**9**	**7.1%**
HCV RNA uninterpretable	2	2.2%					**2**	**1.6%**
HIV								
HIV1/2 assessment	142	87.1%	29	100.0%	7	87.5%	**178**	
HIV1/2 positive	59	41.5%	12	41.4%	5	71.4%	**76**	**42.7%**
HBV								
HBsAg assessment	121	74.2%	29	100.0%	8	100.0%	**158**	**79.0%**
HBsAg positive	6	2.0%	2	6.9%	3	37.5%	**11**	**7.0%**

Anti-HCV = hepatitis C virus antibody; HBsAg = hepatitis B virus surface antigen; HBV = hepatitis B virus; HCV = hepatitis C virus; HIV = human immunodeficiency virus; RNA = ribonucleic acid

Bold indicates total of recruitment methods per subcategory

**Table 2:** Participant demographics

Demographics	*n*	%
Age (years)		
< 25	1	1.1%
25–34	45	51.7%
35–44	31	35.6%
45–54	9	10.3%
> 55	1	1.1%
Total	87	
Mean (SD)	35.5 ± 7.3	
Median	34 [IQR 31–41]	
Sex		
Male	85	97.7%
Female	2	2.3%
Total	87	
Ethnicity		
Black	63	72.4%
Asian	0	0.0%
White	16	18.4%
Mixed ancestry	8	9.2%
Other	0	0.0%
Total	87	

IQR = interquartile range; SD = standard deviation

Following treatment initiation, 84 (97%) participants attended for a 4-week hepatitis C RNA evaluation; *n* = 74 (88%) were undetectable. In total, 75 participants completed all 12 weeks of treatment; 61 (92%) were hepatitis C RNA negative at the end of treatment. A 12-week post-end of treatment assessment for a sustained virological response was performed in 57 participants. A sustained virological response of 90% (*n* = 51) was confirmed. During follow-up, seven reinfections were detected. Overall, 15 (17%) participants missed doses more than 7 days, and 33 (38%) were lost to follow-up beyond 90 days on or after treatment completion. Adverse events (Table 3) were reported in 6% (*n* = 5) including mild gastrointestinal upset, fatigue, or headache. None were noted beyond 4 weeks on therapy.

**Table 3:** Treatment cascade outcomes

Indicator	Target reached (n)	%	95% CI
Baseline			
HCV Viremic	116		
Treatment Eligible	94	81.0%	72.7–87.7%
* Antiretroviral therapy less than 3 months*	*18*	*82.8%*	*9.5–23.4%*
* Anti-HBV therapy less than 3 months*	*1*	*9.1%*	*0.0–4.7%*
* Not Interested in direct-acting antiviral therapy*	*1*	*4.6%*	*0.0–4.7%*
* Viremic Lost to Follow-Up*	*2*	*9.1%*	*2.1–6.1%*
Treatment Initiated	87	91.6%	84.1–96.3%
* Eligible Lost to Follow-Up*	*6*	*6.4%*	*2.4–13.4%*
* Not Interested in direct-acting antiviral therapy*	*1*	*1.1%*	*0.0–5.8%*
4-Week Treatment Visit			
4-Week Treatment Completed	84	96.6%	90.2–99.3%
HCV Viral Load 4 weeks	67	88.2%	78.7–94.4%
8-Week Treatment Visit			
8-Week Treatment Completed	79	94.0%	86.6–98.0%
End of Treatment Visit			
Treatment Complete	75	94.9%	87.5–98.6%
End of Treatment Plasma Specimen	66	94.3%	86.0 – 98.4%
HCV Viral Load Suppressed End of Treatment	61	92.4%	83.2–97.5%
12 Weeks Post-End of Treatment Visit			
12 Weeks Post-End of Treatment Plasma Specimen	57	93.4%	84.1–98.2%
Sustained Virological Response	51	89.5%	78.5–96.0%
Ad Hoc Follow-Up			
Reinfection	7	12.5%	5.2–24.1%
Overall			
Missed Doses < 7 days	15	17.2%	10.0–26.8%
Adverse Events at Any Time	5	6.0%	2.7–14.9%
* Abdominal Discomfort*	*2*	*2.3%*	*0.3–8.1%*
* Reflux*	*1*	*1.2%*	*0.0–6.2%*
* Headache*	*1*	*1.2%*	*0.0–6.2%*
* Sleep Disturbance*	*1*	*1.2%*	*0.0–6.2%*
Loss to Follow-Up > 90 days	33	37.9%	27.7–49.0%

CI = confidence interval; *HBV* = *hepatitis B virus;* HCV = hepatitis C virus

Italic indicates a detailed breakdown of a subcategory

In a post hoc analyses, eight participants who had suspected reinfection rather than delayed 12 weeks post-end of treatment failure had their hepatitis C virus sequenced using Sanger sequencing for the NS3/4A, NS5A and NS5B hepatitis C portion of the hepatitis C genome. In seven participants, no NS5A or NS5B resistance-associated substitutions were observed. In one participant, an NS5A 58P mutation was observed. This mutation is not associated with daclatasvir resistance. We elected to regard this participant as a probable treatment failure. All participants in these post hoc analyses were genotype 1a infected.

Education on viral hepatitis, HIV and harm reduction interventions was offered at each scheduled visit to all participants. At baseline, 67% (*n* = 58) of participants accessed harm reduction packs, 57% (*n* = 50) opioid substitution therapy and 18% (*n* = 16) abstained from heroin use. No new HIV seroconversion was detected at the end of treatment or 12 weeks post-end of treatment screenings in this cohort. At least one dose of hepatitis B vaccine was provided to 93% (*n* = 81) of those screening HBsAg-negative participants with 52% (*n* = 41) completing a full three-dose schedule. No statistically significant difference in treatment outcome was observed, when controlling for all other support services for those on opioid substitution therapy, antiretroviral therapy, receiving harm reduction packs or abstaining from injecting drug use, respectively.

## Discussion

The global elimination of viral hepatitis, and HIV, by 2030 is ambitious. It is likely only attainable once prevention, testing and treatment services are provided to all, high-risk populations. A primary care focus on community-level implementation of services is required to screen, diagnose and rapidly link to care those infected. This can only be achieved with access to appropriate screening tests, diagnostics and antiviral therapy. In South Africa, non-profit and community-based organizations are bridging the HIV care gap in providing HIV testing through flexible and accessible services. Many of these public–private partnerships are vital in assisting government fulfill its constitutional mandate, by responding with rights-based care and thus contributing to a healthier society [26, 27].

We designed a program that would be able to deliver screening and care for hepatitis C at community level to an important at-risk population, PWID. This population is a core promoter of ongoing new hepatitis C infection and requires specific interventions. We aimed to identify new PWID with hepatitis C infection in addition to PWID already known to be hepatitis C infected. We anticipated a high feasibility of tracing beneficiaries already known with chronic hepatitis C registered on the facilities’ database within an approximately 10-km radius of our facility [10, 17]. Furthermore, the program was designed to leverage existing harm reduction services such as opioid substitution therapy programs, with peer support structures, and a call center with a transport unit for the recruitment, retention and support of participants [17, 18]. The primary intent of our program was to assess the feasibility of a decentralized model of care and how it could be positioned in a broader national elimination program for the country. Such programs, in essence, function as micro-elimination programs within high-density hepatitis C populations. Such a hepatitis C treatment program has as yet never been instituted nor assessed in South Africa or the continental sub-region.

Our recruitment and retention in care was significantly impacted by the COVID-19 pandemic, influencing participant mobility due to strict lockdown regulations, displacing individuals into temporary shelters and increasing fear in attending healthcare facilities [28]. During this time, HIV testing and harm reduction services were significantly reduced in all sectors as resources were redirected toward the COVID-19 response [28]. This program responded with a mobile team, in collaboration with primary healthcare providers, to screen, monitor and provide support for PWID. This justified an additional need for rapid diagnostic screening kits and transport to various outreach sites outside of the originally anticipated source population area.

We anticipated an 80% hepatitis C viremic rate, that 30% would be HIV positive and a 2—5% seroprevalence of chronic hepatitis B [29]. The region has a single differentiated antiretroviral therapy program [10, 17]. Our detected anti-HCV seroprevalence was slightly lower than expected for the Pretoria PWID population, based on previous screening data [29]. HBsAg seroprevalence was within a range for a population who likely accessed HBV vaccination after its introduction in South Africa in April 1995. Despite the study team educating participants regarding the self-administration of anti-HCV and HIV rapid diagnostic tests to enhance screening, participants were not comfortable to self-perform these tests without facilitation by a community healthcare worker or peer [22]. [23]

We recognized that several participants recruited were from areas close to the healthcare facility and known to community-based harm reduction programs. We limited this selection bias through opening the recruitment to participants from outside the health district, screening and recruiting in four sub-regions of the Tshwane district, informing a network of local implementation partners about the program and allowing for word of mouth or self-referrals from any socio-demographic background. Locally, less than 10% of PWID are women [4, 17]. Our study followed this trend but enrolled fewer women than anticipated. The underrepresentation is in keeping with other studies sampling the same source population over the same period [30, 31]. We would, however, recommend that special consideration be given to index testing, and increased efforts to reach and include women who use drugs, with an emphasis on gender-specific sexual and reproductive health needs and vulnerabilities [32].

The predominant reason ineligibility for direct-acting antiviral therapy was gaps in antiretroviral therapy initiation and antiretroviral therapy adherence in our participant population [17]. The transient nature of our population made initiating antiretroviral therapy, and achieving stability for 3 months, very problematic. While first being adherent to and clinically stable on antiretroviral therapy are not contraindications to hepatitis C therapy, several factors underpinned our protocol. We have a background prevalence of tuberculosis; thus, antiretroviral therapy stability for 3 months prior to direct-acting antiviral therapy helps eliminate complications such as tuberculosis immune reconstitution syndrome. It also provides an opportunity to engage participants in therapeutic adherence prior to direct-acting antiviral therapy being initiated.

A significant unexpected practical issue that influenced sample workflow was difficulty in venesections for whole blood draws in a PWID population. This affected sample processing using the Genedrive® assay, with practical troubleshooting measures being required. The Genedrive® yield of uninterpretable results, primarily due to hemolysis, was improved with a plasma dilution protocol and in-service quality improvement interventions for phlebotomists [25].

Overall, Genedrive® performed well as a point-of-service assay to seamlessly and more rapidly link participants to care. We have demonstrated that in this challenging patient population, traditional models of facility-based care will not achieve the scale-up and treatment impact required to eliminate hepatitis C in this population. The traditional standard of care encompasses several visits at primary and specialist level, costly assessment and invariably, limited adherence support and access to harm reduction [15]. Furthermore, case-finding is limited and viral hepatitis screening not necessarily prioritized [29]. Although the study was limited by the lack of direct comparison to the traditional model of care within this cohort, we believe our results demonstrate the feasibility of our point-of-service model. Some practical challenged could be addressed by innovative solutions such as dried blood spot to assess for qualitative hepatitis C viremia and/or quantitative hepatitis C RNA [23]. The current infrastructure and skills within our antiretroviral program, with differentiated care for people who use drugs, should be leverage alongside advances made with Project ECHO clinics support [33].

Our participants achieved a 90% sustained virological response rate. A rate of ≥ 90% for direct -acting antiviral therapy, is regarded as acceptable and a benchmark for treatment [15]. Although we observed that uptake of a single harm reduction intervention such as opioid substitution therapy, antiretroviral therapy, harm reduction packs or abstinence can benefit, further research is needed to ascertain best practice for different harm reduction interventions in the South African context [8]. Reinfection is well described in the PWID population, the reasons equally known [34]. Our confirmed reinfection participants all had inconsistent access or uptake of support services, for example, the accessibility of opioid substitution therapy services, which is associated with a reduced incidence of reinfections, due to a lack of general availability of opioid substitution therapy, cost and the lack of implementing a national policy. These factors require dedicated attention, prioritization and the allocation of directed resources [29, 35, 36]. During serial HIV screenings, no seroconversions were detected in this high-risk population. This emphasizes the need for domestic structures to prioritize access to harm reduction interventions at community level, which have been shown to reduce the incidence of HIV and other blood-borne pathogens [36].

A decentralized patient-centered harm reduction strategy, to screen and link PWID to care for HIV and viral hepatitis at a community level, should be scalable in the major metropolitan areas of South Africa. What is required is a simplified tracing model that includes community- and peer-led outreach campaigns, with collaborative partnerships for treatment support and effective inter-program referrals. Population-sensitive and cost-effective [37] hepatitis C treatment through community-based full point-of-service care is attainable in conjunction with social and harm reduction support services, in a resource limited setting, for this key population. However, sustained access to harm reduction services remains dependent on unified, multisectoral, evidence‐informed strategies at political and technical levels to attract and sustain commitment and financing [11].

## Conclusion

A simplified point-of-service model for PWID with chronic hepatitis C virus infection yielded an acceptable sustained virological response rate in our setting. Retention in care and follow-up remains both challenging and key to outcome. Our model provides a basis for other countries in our region to utilize this more community acceptable and simplified practice.

## Data Availability

The datasets used and/or analyzed during the current study are available from the corresponding author on reasonable request.

## Abbreviations

Anti-HCV: Hepatitis C virus antibody
CI: Confidence interval
UI: Uncertainty interval
COVID-19: Coronavirus disease
DNA: Deoxyribonucleic acid
DAA: Direct-acting antiviral
HBsAg: HBV surface antigen
HBV: Hepatitis B virus
HCV: Hepatitis C virus
HIV: Human immunodeficiency virus
IQR: Interquartile range
PWID: People who inject drugs
RNA: Ribonucleic acid
SAHPRA: South African Health Products Regulatory Authority
SD: Standard deviation
SOF/DAC: Sofosbuvir/daclatasvir
